# Memorials of the Faculty of Physicians and Surgeons of Glasgow, 1599—1850

**Published:** 1896-12

**Authors:** 


### Reviews of Books.

**Memorials of the Faculty of Physicians and Surgeons of Glasgow, 1599—1850.** By Alexander Duncan. Pp. xiii., 307. Glasgow: James Maclehose and Sons. 1896.

Histories, even when accurate, are wont, unless written by a scholar, to be at the best dull reading; but Mr. Duncan, the Secretary and Librarian of the Faculty, has in the book before us compiled an interesting and eminently readable work on a subject of which he is evidently complete master.

It has been a source of great pleasure to us to read the *Memorials* of this, the oldest medical corporation in Scotland, and not only is the author to be congratulated on the completeness of his work, but the Faculty also on having so able an historian to tell them all about themselves in the past. The Faculty obtained its charter from James VI. on November 29th, 1599, at the instance of Dr. Peter Lowe—whose handsome portrait forms the frontispiece to the book—and Robert Hamilton; and, unlike the subsequent foundations of a similar character, had powers (which it used) over a considerable area of the surrounding district to examine, to license and prohibit practice, and to amerce a fine by "letters of horning." The granting of a charter of this nature to a profession in a town of only 7,000 inhabitants is certainly a very remarkable event, and it is only natural that the powers given the medical profession were not amicably received by the civic authorities of the town. As the author points out, it was probably due to Dr. Lowe and his foreign experiences that the barbers, then a branch of the surgical side of medicine, were given such an inferior position; for they were admitted as a "pendecle of Chirurgerie" only as a matter of grace, and were allowed "to midle not w[t] anything farder belonging to Chirurgerie." Even lithotomists, or "stone cutters," were only admitted on sufferance, the Faculty viewing the operation as "too dangerous a procedure ... to rank with the ordinary practices of the life-preserving art." They were therefore appointed by the magistrates and paid out of the rates; but occasionally, as in the case of Evir M'Neill, who cut for forty-two years (1646—1688) and whose signature was made by a notary, they were admitted on account of their skill and for services rendered.

All through the book there is evidence of the jealousy of the Faculty in regard to any interference with their rights, and of the persistence with which they fought for the privileges conferred on them in the charter.

We wish we had space to refer to the many more interesting facts we have come across in reading these *Memorials*, but must content ourselves with recommending the student of medical history to read the book for himself. Among the most distinguished alumni were Dr. Peter Lowe, Robert Houston, the father of ovariotomy, William Smellie, the obstetrician, William Cullen, John Moore, the father of Sir John of Corunna fame, and Dr. Joseph Black, the discoverer of carbonic acid gas.

We cannot conclude this notice without hearty praise to the publishers for the general appearance of the volume. The type and paper are most attractive.

